# The Strengthening Mechanism of the Relationship between Social Work and Public Health under COVID-19 in China

**DOI:** 10.3390/ijerph18199956

**Published:** 2021-09-22

**Authors:** Guanghuai Zheng, Xinyi Zhang, Yean Wang, Mingzi Ma

**Affiliations:** 1 School of Sociology, Central China Normal University, Wuhan 430079, China; zhenggh@ccnu.edu.cn (G.Z.); shxyzxy@mails.ccnu.edu.cn (X.Z.); 2 School of Social Development and Public Policy, Beijing Normal University, Beijing 100875, China; Echowang@bnu.edu.cn

**Keywords:** health social work, public health, legitimacy, COVID-19, China, relationship

## Abstract

Social work and public health have always shared a common mission and vision in promoting human health. However, existing research tends to view social work and public health as two separate fields at both practice and policy levels, and these studies have largely neglected the consideration of how to integrate public health and social work. In the context of the COVID-19 epidemic, the link between the two has been strengthened and health social work has been given more importance. The question addressed in this article is through what mechanisms or practices the social work profession can strengthen its professional status and engage in interprofessional collaboration. Based on key informant interviews and case studies (one community and two cabin Hospitals), this study points out that three legitimacy mechanisms are needed: operationalizing policy, extending value, and completing justification. Furthermore, the future and possible limitations in relation to the development of health social work in China are discussed and specific recommendations are provided. Health social work needs to conduct practices and summarize its experiences and methods, to create a more friendly political environment by translating its results into policies that are conducive to the development of health social work through a political agenda. It needs to improve upon its practical abilities and methodologies, as well as professional education relating to professional values and ethics, in addition to identifying the deeper social needs of residents and discovering new, undeveloped areas of service. Moreover, because long-term change is difficult to justify due to China’s policy agendas, the question of whether the professional status of health social work in the post-epidemic context can be improved is something that needs to be further explored in future studies.

## 1. Introduction

The outbreak of COVID-19 has impacted people’s work and lives in many ways [1]. People cannot stand aside, as they do in the face of other disasters or diseases—everyone is required to act in order to respond to the outbreak and to prevent its spread. Many professional groups have taken action, including doctors, nurses, community health workers, and social workers, and that combined effort is the focus of this article. Social workers around the world have taken many impressive actions to ensure the survival and dignity of humans, to address structural inequalities that may be growing in the context of the epidemic, to provide mental health counselling for the general population, to solve the problems of unemployment and domestic violence, and so on [2]. However, it seems that social workers are rarely considered as human resources in the field of public health.

In China, a series of top-level programs, such as the Ministry of Civil Affairs and the National Health Commission’s [3] jointly issued “Urgent Notice on Further Mobilizing Urban and Rural Communities on the Prevention and Control of Pneumonia Outbreaks Caused by the New Coronavirus”, have emphasized the role of social work in epidemics and public health and have to some extent helped to increase the professional status of social work in China. Furthermore, the interaction of social work with the public health system has been significantly enhanced. The evolving situation in regard to the changes and practices in the social work profession worldwide and China in the context of the epidemic has inspired us to revisit and explore the following issues: how social workers are becoming part of public health and identifying the mechanisms by which to strengthen the collaboration between the two.

### 1.1. Social Work and Public Health

A professional group within the social work profession has also begun to regain its interest in public health in recent years [4]. Social workers have a complex identity, with knowledge in psychology, sociology, and medicine, and they are not only members of the social sciences but are also health professionals who on the one hand play an important role in the clinical field, and on the other hand, have a shared interest and responsibility in promoting health equity and improving the social determinants of health and the conditions in which people live [5]. Social work has long played an important role in promoting human health and well-being, and in that light, all social work can be regarded as health social work [6,7]. Social work is ideally positioned, with its unique professional values and ethics, but as noted, current social workers may be too focused on clinical practice, while ignoring “social factors” [4]. Therefore, the branch of social work known as public health social work was born. Public health social work links micro- and macro-level social work with social epidemiology, disease prevention, and health promotion [8], using multiple perspectives and methods to solve significant and vital health issues, to promote health equality [9], and to provide the overall environmental conditions for a healthy life [10].

Ruth et al. described the relationship between the levels of health social work and their interaction with health systems through social work, using a health impact model [6]. From the most important clinical interventions to a subsequent broader focus on factors that can affect population health, the three dimensions of prevention, policy practice, and broad social activism have all become areas that should be the focus of health social work. On a micro-clinical level, consider the medical system, for example. Medical social workers can act as patient navigators to help patients obtain the help they need in a complex healthcare system [11]. This role involves helping patients to comply with physicians’ treatment recommendations, regulating the doctor-patient relationship, and easing patients’ emotions [12,13,14]. The meso level focuses on community-level prevention and health promotion, such as designing community-building activities that promote collaboration among community members [15], directing investment and collaboration based on multisectoral partnerships [16], and providing additional healthcare facilities and the information that patients need about healthy living environments and treatment decision-making options [16,17]. At a macro level, health social work aims to enable changes in the systems and social factors that influence health [6], such as by demonstrating the social determinants of injustice and its associations with people’s health [18], using a variety of methods for structural interventions across sectors to promote changes in public health policy based on that evidence [19].

The role of health social work in response to public health events such as infectious disease outbreaks can be seen as an integration of the three dimensions (micro, meso, and macro). For example, in the Ebola epidemic in Africa in 2014, the interventions provided by local health social workers included providing necessary psychological support at the individual level, carrying out knowledge and publicity activities at the community level to improve community members’ knowledge about epidemic prevention, helping marginalized communities to obtain basic medical services, and providing necessary services for groups that were stigmatized due to the epidemic [20,21]. Therefore, health social work can play an important role both generally and in special targeted situations.

### 1.2. Health Social Work: Its Practice in China

In China, we must acknowledge that the tradition and approach of combining public health and social work have been gradually accepted and used in recent years, but the links between them are still very limited. Researchers have gradually explored the impact of social factors on people’s health, but these concerns are relatively primary, and studies have focused mainly on specific groups. For example, Tong and Lai explored the relationship between the physical and mental health of older adults in urban China from the perspective of social exclusion [22] and Wang et al. pointed out that ‘left-behind children’ are the result of socially constructed inequalities [23]. However, for those who pay attention to macro factors, a connection is lacking between research and actual practice and intervention, with studies rarely or only symbolically having mentioned possible intervention measures—whether they were policy or clinical measures [23,24]—and seldom having mentioned the role and function that social work may play in public health.

Second, the practice of health social work in China has limitations. In China, with its medical system reforms and the transformation of the traditional medical model into a social-psychological-physiological model, health social work has been only gradually introduced into the current medical and health care system [25]. The early aim of the development of health-related social work was to improve patient–doctor relationships in violent cases, to safeguard the legitimate rights of doctors and patients, and to enhance medical efficiency and quality [14]. Beyond that, social work also has participated in the case management of patients with major diseases and hospice and palliative care [26,27]. In recent years, mental health social work has also been gradually recognized by the academic community and the state [28]. However, the introduction of the field of health social work was recently only micro-oriented and was deeply embedded in the current medical system and institutions [29]. The above studies only describe the temporal situation of health social work in China, and do not address the mechanisms of how to strengthen the link between social work and public health.

During the COVID-19 epidemic, however, social workers in China have been active in the broader field, such as in mental health and community-level social work, which have also been mobilized to enable social workers to participate in all stages and processes of the treatment, response, and prevention of the epidemic. This has opened a window of possibility for exploring how the profession can move toward the broader field of public health in the future. After conducting a comparative study before and after the COVID-19 epidemic and basing our investigation on the concept of the legitimacy of neo-institutionalism [30], we found that mechanisms showing how major events affect the reevaluation of professional legitimacy by different groups may be crucial to strengthening and facilitating the collaboration between social workers and the health care system. Thus, we propose the roles of three legitimacy mechanisms.

## 2. Conceptual Framework: Professional Legitimacy

In this article, we have attempted to put forward the exploratory concept of the image of legitimacy to investigate the change in the professional status of social work within the epidemic context. First, legitimacy is the key issue in relation to changes in professional status. The concept of “legitimacy” originated from Max Weber, who believed that the legitimacy of a certain political regime lies in the participants’ beliefs and faith [31]. Altshuler and Webb pointed out that a legitimacy crisis is caused by the unclear definition of role expectations and the professional/educational requirements related to state certification [32]. Warner proposed that legitimacy may be addressed in three domains: new knowledge based on shared value, institutional arrangements, and conditions for social work practice [33]. In studies on Chinese social work, Wu Lei pointed out that the improvement of legitimacy is based on a profession’s effectiveness [34]. Tong and Zhou argued that legitimacy includes not only the issue of professionalism, but also whether the profession and its services could be recognized by relevant service objects and stakeholders, that is, the perspective of “recognition”, because although many welfare professionals have completed their work with high quality, they have not received a corresponding reputation, and the lack of a proper identity leads to an inability to carry out more professional work [35]. According to the above studies, we believe that in order to make social workers a new human resource in the field of public health, we need to improve their legitimacy from three aspects.

First, the legitimacy of social work can be promoted by the existing laws, policies, and institutions (that is, the dimension of legality), and thus legality constitutes an important dimension of the legitimacy of social work. Legality represents a kind of policy compulsion [30], referring to governmental cognizance and institutional arrangements, which together form the premise of the formal development of social work.

Second, the legitimacy of social work can be promoted by responding to practical problems. In this context, the value of a professional practice is determined by its ability to respond to the urgent needs of people and to solve problems effectively [36]. Value focuses on the professionalism and effectiveness of social work services in specific scenarios. Social work can be promoted only when it demonstrates significance in a specific scenario and thus can respond to major practical problems.

Third, because health social work is an “imported” profession, our understanding of its functions, effectiveness, and knowledge system is deepening gradually. Moreover, because the development of social work in China is at an early stage [37], the profession has had to wait to earn increasing recognition over time, especially the recognition from its cultural system [38]. Recognition is a manifestation of justification. Justification refers to the ethical codes and moral standards that are generally accepted by society, and it is a key dimension that social work must master in order to gain the understanding and recognition of all related subjects and to be regarded as engaging in reasonable and common professional practices [39]. Only when social work is widely understood and acknowledged by the public can its functions and interventions be broadly accepted.

In the Findings section, we will show how social workers have gradually gained legitimacy in public health-related fields.

## 3. Methodology

In this study, key informant interviews and case studies provided opinions on the nature of the problem of health social work as a profession in flux and helped us to propose solutions that were based on the special knowledge and understanding of the interviewees [40]. The study received ethical approval from the ethics committee of the School of Social Development and Public Policy at Beijing Normal University in China (reference number: SSDPP-HSC2019003). Before the outbreak, the researcher participated in internships at some of the interviewed hospitals and used the internship sites as the primary study cases. After the outbreak, a community in Wuhan and two cabin hospitals became the cases of interest in this study. Some of the practice experiences and models mentioned in the Findings section were also drawn from these cases. At the same time, the workers in the cases were also our interviewees.

During the two periods before and after the outbreak of the COVID-19 pandemic, 21 interviewees were ultimately selected (categories and quantities are shown in Table 1). All interviewees were informed about the objective of the study and did not have a patient status. The interviewees had all worked in the social work discipline for more than five years. The reasons for selecting these interviewees were that the government officials had been directly involved in the policy-making agendas of public health and social work, the interviewed experts and researchers had served as government advisors and the main leaders of related research programs, and the rest of the interviewees were experienced public health and social work workers or managers.

The interviews were conducted face-to-face, by telephone, and via the Internet. The researcher audio-recorded all of the interviews and hand-wrote some of the interview notes. Table 1 shows the different types of respondents and their numbers in the overall sample. At the same time, the researchers observed some field practices. The semi-structured interviews mainly consisted of open-ended questions in the Chinese native language. The questions were identified in cooperation with experts from the target population to ensure their appropriateness and effectiveness. The core questions for the respondents were the same, but adjustments were made on specific issues according to their actual situations and backgrounds. Here are some examples of specific questions:

- What is the current organizational structure of the Social Work Department? If there have been changes in the development process, have there been any impacts on you before and after the changes?
- Do you think that the current professional development program, as far as it is concerned, can meet the professional needs of health social work?
- During this epidemic, in your opinion, how did social workers perform, did they respond to the needs and expectations of the community, and to what extent did they respond? In the future, is it possible for social workers to obtain preventive content when responding to possible epidemics or other social events?
- What are your views on the current and future development of health social work as a whole? What do you think are the factors that affect the development of health social workers?

Analyses were conducted by the researchers in the following specific steps. First, interviews were recorded and transcribed. The transcription process was completed by researchers, and after combining the transcribed content with the observation notes, subthemes were determined around the general themes of the current level of social work legitimacy changes in the context of the epidemic.

Secondly, to avoid ambiguity and misunderstanding, the researchers retained the language of the interviewees in the presentation of the data and only optimized unnecessary content and semantic content. Then, the researchers classified and recoded the content of the interviews according to the theoretical framework. A thematic analysis of the data was conducted to look for recurring dimensional themes related to legitimacy. To ensure reliability, the research team read the data line by line and developed a coding scheme to represent the core themes in the data for repeated or similar content [41], and representative content was selected after discussion and consultation. As needed, the statements of nine interviewees were eventually quoted directly.

Finally, the research results and explanations were written into preliminary findings, which were reviewed and finalized by all team members involved in the study’s conception and actual research. Following that review by the team members, the researchers introduced the preliminary findings to certain experts, who helped to improve the final interpretation. These experts included some of the interviewees in this study, as well as professors from social work departments in other universities. In addition, the unfinished first draft and conclusions of the study were reported at an academic conference in China, where they received public comments from peers.

## 4. Findings: Three Mechanisms of Health Social Work’s Legitimacy Image

### 4.1. Operationalizing Policy

The operationalizing policy of legality refers to the process of the gradual refinement and operability of the original difficult-to-implement or contradictory policies and regulations. The legitimacy of social work will be strengthened by more explicit regulations and measures, more government support, and resource investment. The government is the main builder of legality. Only within the framework of legality can professional status be established and promoted.

Before the COVID epidemic, the legality of health social work was in a state of gradual development on the macro level and stagnation on the micro-level. The gradual development of the legality of health social work on the macro level was reflected in the gradual introduction of policies and documents. In 2009, the “Opinions of the CPC Central Committee and the State Council on Deepening the Health Care System Reform” began to regard health social work as an approach that could be used to solve non-medical health social problems. In 2013, the National Health and Family Planning Commission further expanded health social work to medical charity assistance to better carry out medical treatment work for disadvantaged groups. In 2016, the National Health and Family Planning Commission and 22 other departments jointly issued “the Guidance on Strengthening Mental Health Services”, which further clarified the function of health social work in providing mental health services in the aftermath of public emergencies, as well as recovery and reconstruction. In 2017, in the “Action Plan for the Further Improvement of Medical Services” (2018–2020), medical institutions were required to establish a system of medical social workers and volunteers from the perspective of top-level design for the first time.

However, differences between the civil affairs system and the medical and health system to the development of health social work have caused its legality to become stagnant. The interviewed leaders of the Civil Affairs Department thought that health social work was paid attention to because it was the best breakthrough point to reflect the professionalism and social value of social work.

“Social workers, to be honest, are not so professional in all fields. But for example, the field of health social work is a field that can significantly reflect the professional level of social work. The area that can reflect professionalism is what we want to further promote through policies.”

(Policy, P2)

The Medical and Health Management Department aims to spend more of their limited energy and resources on improving the quality of medical services and technical capabilities, and the expectations on social workers were also focused on this. Medical and health management departments have not recognized the expectations of civil affairs departments to highlight the professionalism and social service value of health social work. Due to the lack of consensus among government departments, health social work has stayed at the conceptual level and there has been a lack of promotion of specific systematic and guiding policies. As an official from the Civil Affairs Department said:

“In the opinions issued by the 12 departments in 2016, it has been made clear that social workers should be set up in hospitals in the medical field, and the health and Family Planning Commission is also a countersignature unit, which should be supported at the policy level. But for more specific policies, at present, there is no deeper promotion.”

(Policy, P2)

As Professor A1 from a university said,

“The priority now is to make the leaders at the national level clear about the specific role and importance of social workers in the medical system.”

(Academic, A1)

The legality of health social work is recognized more on paper than in real-life practice, raising the dilemma of a lack of jobs and funds at the specific level.

Within the context of the COVID epidemic, the coordinating role of health social work between communities and hospitals was highlighted. The establishment of a social-worker-led hierarchical defence model between communities and hospitals in Wuhan alleviated the serious shortage of hospital beds to a certain extent. The government began to realize that only by operationalizing the legality of health social work, could health social work really become a bridge between the community governance system and the public service system, such as healthcare providers. This understanding was fully reflected in several policy documents and work guidelines issued by civil affairs and health departments after the outbreak. Among government documents, the most important one was issued by the National Health Commission and the Ministry of Civil Affairs. This “Notice on strengthening psychological assistance and social work services in response to the COVID-19 outbreak” not only clearly pointed out the scope of work and service objectives of health social workers during the epidemic, but also published three specific work programs: “Psychological assistance and medical social work service program in novel coronavirus pneumonia treatment hospitals”, “Psychological assistance and medical social work service program in cabin hospitals” and “Psychological assistance and medical social work service program in novel coronavirus pneumonia prevention and control centralized isolation points”.

Before the epidemic, although the concept and function of health social work had been initially established and accepted, its legality had not been further specified. The government financial support had not been provided in a sustained manner, and it could only be seen in sporadic project purchases. Due to the lack of more operational and specific provisions and guidance, health social work lacked detailed legality, and could not be carried out in hospitals or communities. The outbreak of the COVID epidemic started the mechanism of legality. People realized that the abstract and unclear legality limited the important function of social work. Then, the legitimacy image changed, which urged the government to quickly operationalize the policies and documents for health social work through “reactive legislation”.

### 4.2. Extending Value

Expansible value refers to the process of a professional field expanding outward in content and function, showing the value that has been ignored or not shown, and then promoting the professional status.

The aim of introducing social work into the health system was to relieve tense doctor–patient relationships and promote communication and understanding between doctors and patients. However, in fact, when problems such as death and huge compensation arise in medical disputes, social workers can do very little and have no special advantage compared with administrative staff. As HC2, a medical social worker at C hospital, said:

“If I want to deal with the medical disputes, I may think about whether social workers can play a role from a lot of professional perspectives. Of course, I find that they do not seem to play many roles, because I find that when dealing with medical disputes, you will be more effective if you have experience. I find that many experienced executives will do better than our social workers.”

(Hospital, HC2)

At the same time, in the hospital field, social workers need to prove their professional value through various means to avoid being replaced by other professionals. For example, HG1, the leader of G hospital, clearly pointed out that:

“Social workers in the hospital are awarded as Professional and technical positions like doctors and nurses, not administrative positions. We need to prove our professionalism, including the scientificity and preciseness of our services. We need to show ourselves in some ways like scientific research.”

(Hospital, HG1)

Health social work in hospitals must develop similar scientific research abilities in order to get the attention of the hospital. The problem is that it is difficult for social workers to prove their professional value as doctors do through their scientific research abilities, because this requires strong medical or health-related abilities, which the existing social work education system cannot provide. In the hospital context, it is difficult for health social work to reflect its value by improving its scientific research ability.

However, despite the failure of social work in the hospital, it has achieved success outside the hospital. The epidemic situation provided an opportunity for the value of health social work to expand rapidly. In addition to hospitals, temporary cabin hospitals were also important anti-epidemic places. Although the government rapidly built cabin hospitals and treated many patients, it lacked the resources to provide patients with services besides basic medical care. Similarly, the governance system of grassroots communities at that time was far from being able to cope with the public health needs of concentrated epidemic outbreaks. Therefore, the government, medical staff, patients, and community residents were looking for another resource to help improve the current situation. The services provided by the cross-professional team led by Professor W1 and other social workers in Wuhan cabin hospitals are good examples of the expansible value of health social work.

“The most important characteristic of the cabin is that the patients in the cabin have a very high demand for order. The number of medical staff is very limited, they are too busy to do all the things… In short, the management of the cabin hospital is very chaotic. Our team provided management and support, immediately reduce much work of medical staff, and the whole order becomes a lot smoother. For example, some hygiene or common-sense questions can be answered by our social workers… There is also psychological crisis intervention, we will deal with the case immediately… Later, they had a high degree of trust in us, including hospitals, which issued notices and handled discharge certificates through our online community.”

(Academic, W1)

Furthermore, the community, as an important place for epidemic prevention, faced many difficulties and challenges that were rarely encountered in ordinary times, which also provided a space for health social work to demonstrate its value. The “2 + 3” online community anti-epidemic model, developed by a Wuhan social work service team, was another attempt to expand the value of health social work. “2 + 3” meant that two professional workers (social workers, community workers) and three volunteers (medical volunteers, psychological volunteers, administrative assistants) cooperated with neighborhood committees and relevant medical institutions to establish a “community-based” three-tier prevention mechanism using Internet tools. The team entered multiple communities and isolation points through the Wechat group to carry out online counselling and appeasement medical consultations and crisis intervention services.

Before the epidemic, health social work had difficulty responding to the actual needs of the community because of a lack of ability and the inappropriate and unmatched social work education in China. With the outbreak of the epidemic, there were more dimensions of needs to be met within hospitals and society. The original content and scope of health social work could not meet the actual needs of the epidemic, thus forming a negative legitimacy image at the value level. Facing this negative legitimacy image, the social work profession took action to respond to social needs under the epidemic situation, and re-expanded and responded in many aspects, such as work content, methods, and places. Although these works were not all “professional”, they built their own professional value through their own efforts, professional knowledge, and ability.

### 4.3. Completing Justification

Completing justification refers to the point at which a profession is recognized by other professions and society because it can play a unique role that those other professions or occupations cannot replace. As mentioned above, with the legality promoted by the government, the concept and role of health social work were accepted and recognized to a certain extent. However, in practice, the awareness of patients and medical staff about health social work (medical social work) was mostly confined to the role of non-professional and replaceable consultants or volunteers, akin to a kind of “senior nursing worker” without charge in a hospital.

“In our hospital, social workers mainly provide patients with some information about medical expenses; when patients are hospitalized, they provide admission procedures, location guidance of various departments in the hospital, and information about attending doctors …. There are also some recreational activities and volunteer services, which are the most popular among us (doctors) and patients...”

(Hospital, HD3)

Health social work did not demonstrate its indispensability in this context. First, although the service objects (such as patients and doctors) recognized their services, they did not have a high degree of recognition of their professionalism but regarded it as work that ordinary volunteers or enthusiasts could complete. Second, although some hospitals had set up health social work (medical social work) positions, most of these positions were held by nurses in hospitals, which formed an idea among medical staff and patients that health social work was an expansion of nurses’ responsibilities, and its professional status was therefore dispensable. Third, although health social workers played the role of resource linking, such as carrying out medical assistance projects for the poor, which could better show their professionalism, these services were relatively concentrated in some specific service places (such as only in some large and famous hospitals), which made it difficult for health social workers to obtain the awareness and recognition of more extensive service objects. Hospital management and front-line medical staff generally rejected and disagreed with social workers, and even refused to cooperate with health social workers.

“Some hospitals are doing worse, and there is an extreme situation, that is, they do not care about anything…I entrust you (social workers), then you do it. Anyway, we do not understand any knowledge of you…Social workers must cooperate in many aspects, but cross-professional cooperation may be very difficult.”

(Organization, O3)

Low cognition made it difficult for health social work to find a foothold to carry out services in hospitals, and it was also difficult to achieve cross-professional cooperation with medical staff. As time went on, it could not exert its due influence. This, in turn, limited the function of social workers, who were mostly responsible for processing documents, copying medical records, carrying out patient satisfaction surveys, and other auxiliary work.

The outbreak of the epidemic highlighted the irreplaceable uniqueness of health social work in the cross-professional team, and the cognation was greatly improved. In the cabin hospital, at first, social workers composed of external third-party teams wanted to enter the cabin hospital online to provide services, which was refused. After that, the team proved its professional value by providing a series of services and was finally recognized by medical staff and patients in the cabin hospital. As Professor W1 said:

“Maybe society has no expectations for professional social workers because many places don’t know what social workers are. But at least this time we Let a lot of people know what social workers are… When we first entered the cabin, the medical staff were also very suspicious, but we relied on professional services to let the medical staff and patients recognize it. If we still doing something Worthless, they will not have social work in his mind, and they don’t know how to use us (social work).”

(Academic, W1)

The cognizance of health social work was not only reflected in the cross-professional team but also reflected at the community level. During the epidemic period, for the general community residents, social workers mainly carried out services through offline means, such as:

“Asking about the physical condition of the residents, taking their temperature, doing a good job in registration, sending masks and distributing epidemic prevention manuals.”

(Organization, O4)

For the four specific groups (mild confirmed patients, suspected patients, fever groups, and close contacts) who required home isolation or centralized isolation, social workers mainly provided online counselling, emotional relief, psychological support, and other services to relieve the patients’ tension and anxiety. The social work service not only strengthened the close interaction with residents in the short term, but also caused residents to experience the indispensability of social work in daily life, and further strengthened the awareness of health social work. All these fully demonstrated the unique role of health social work in the epidemic context.

Although the legitimacy image of healthy social work was in a low state before the outbreak, it is still positive at present, which means that people are satisfied with their expectations of the ability and role of health social work. Therefore, before the epidemic, health social work was easily limited to specific service places or service items, which made it difficult to be fully recognized by medical staff, and it was also difficult to establish and strengthen its legitimacy at the community level. The arrival of the epidemic broke the balance. As all members of society were facing the public health crisis, the services provided by the field of health social work for the entire society proved its indispensable role in this special period. For the public, medical staff, government officials, and community workers alike, their attention and demand for health social work reached an unprecedented height, which jointly promoted the professional status of social work.

## 5. Discussion

### 5.1. Legitimacy as a Mechanism

**Figure 1:**
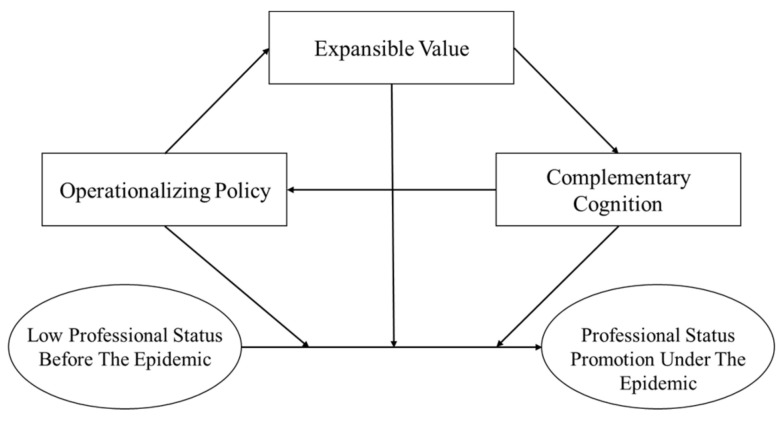
The mechanism of the promotion of the professional status of social work.

The concept of a legitimacy image reveals that only when the relevant subjects re-consider and reevaluate the level of correspondence between the legitimacy of different dimensions and practical experiences can professional status can be improved (see Figure 1). In regard to legality, the specific guidance documents issued by the central government directly emphasized the importance and functional position of social work. Therefore, policymakers (for example, local government) who did not pay attention to or thought that developing social work was not urgent [38] had to re-examine its professional status. The operationalized legality reduced the resistance of social work and promoted social work to obtain more resource support and grassroots recognition and enabled it to provide services more generally. More independent legal and policy protection and financial support can improve the social power and status of welfare professionals or related organizations, allowing them to better develop and perform the role in the provision of welfare [42]. In the value dimension, the urgency of the epidemic created expectations from the government, other professional groups (such as medical staff), and the public in relation to social work. The practice of social work in shelter hospitals and other new venues responded to external doubts about its professionalism and concerns about its effectiveness within the context of the epidemic. The reevaluation of this practical experience and the value of social work by relevant subjects has promoted its professional status. Third, completing the justification of social work as a profession changed people’s cognizance of its supplementary and auxiliary status. Now, more and more people regard social work as an indispensable profession in the provision of health services.

### 5.2. The Future and Limitations for the Professional Development of Health Social Work in China

On the practical level, the concept of legitimacy reveals how social work can use the “opportunities” in the context of major emergencies to further link itself more closely to the health profession. At present, the field and places of social work mainly depend on the adjustment of national macro policies, the transformation of governmental functions, and institutional reform [43]. Professional development is reactive, with little awareness and discourse coming from the profession itself. Medicine and social work are inseparable and share a common position and commitment to human health and social justice [4], but Chinese medical practice in recent decades has traditionally focused on medical treatment more than on prevention, and the medical system and the public health system were almost two separate systems until the outbreak of SARS [44]. Moreover, social work has not established a foothold in either field, although the practice of social work before the epidemic had established links to the field of health. On the one hand, in hospitals, social workers are used to regulating doctor–patient conflicts and performing some types of rehabilitation treatment, but on the other hand, social workers in China are engaged in various fields related to the survival and health of the population. Examples include their role in the prevention and treatment of AIDS in rural areas of China and in reducing social exclusion and providing social support for vulnerable groups in rural areas [45]; the role of social work for the rehabilitation of drug users in urban communities [46]; and social workers’ roles in community rehabilitation after disasters such as earthquakes, promoting equity, helping vulnerable groups to obtain the necessary food and medicine to ensure the health of survivors, and the like [47]. However, just as Ruth and Marshall found when evaluating social work in the United States [4], China also clearly has similar problems: although social work plays a role in various fields and systems, it lacks integration, and it is unable to clarify the relationship between itself and the current public health system.

In the Health China 2030 policy, the government emphasizes prevention, the decentralization of communities, and improving the quality of living environments, such that everyone will have access to comprehensive healthcare services throughout their entire lifetime [44]. However, the policy also faces many challenges. Who should carry out this policy—doctors or social workers? Many questions are still waiting to be clarified. Wong argued that government reform offers great opportunities for health social workers to move from being hospital-centered to community-based [48], but social workers still face many challenges, such as their education and the challenge of working with different models of collaboration with governments and other professions [49]. Chinese social workers’ practices in the context of the epidemic have tapped into the true potential of social work in public health, and although the workers may still be more clinical in their services at this time, a space for community-based interventions has been created, and policies, experiences, and practices have been established. Therefore, social work’s use of major events to proactively connect with the health field should also be a lesson learned from this outbreak. Realistically, however, scholars still criticize social work for not being formally integrated into the public health response system in the aftermath of an outbreak [50]. In addition, the health challenges of economic inequality, environmental pollution, and the like, are still far-reaching and difficult, and it remains to be seen whether social work can integrate itself and practice in these areas in the future. One bright prospect is that the new public health policy plans to seize the opportunity to work with more partners, especially non-health-sector players to address issues at the policy and structural levels, not just at the individual level [44]. Social work, especially health social work, should be expected to be well-positioned to build a new bridge between the social sciences, medicine, and public health.

However, another important issue is that the development of health social work in China may also have the following constraints.

First, due to the wide existence of departmental politics, it is difficult to predict whether the promotion of social work in the epidemic context can be transformed into a new breakthrough in policy. After the prevention and control of the epidemic had become part of people’s daily lives, the National Health Commission and other relevant government departments issued a series of documents stating that health social work should be introduced into more fields. However, these fields have continued to repeat the emphasis on the original fields of social work and have failed to really provide an integrated policy framework. The reason for this may be because the internal containment or linkage among government departments leads to disorder and incoherence in the policy system itself [51]. The civil affairs department and other departments cannot reach an agreement on the status of social work in the institutional structure. Even if a superficial agreement is reached, it cannot be implemented in a specific context, such as transferring the corresponding resources and rights and maintaining the independence of social work organizations [52].

Second, to have value in the field of public health, social workers still generally lack sufficient relevant knowledge and practical experience. Social work in China is based primarily on the requirements of grassroots organizations, governments or the relevant bureaucracy, and previous experience. Although social workers do play an important role in psychological assistance, the protection of children, and providing services to the elderly, credible clinical service models and justice- or rights-based service orientations are still lacking. This type of value is more reflective of the value of auxiliary grassroots bureaucrats and administrative systems than its professional value.

Finally, health social work still faces the problem of how to position itself within the current state–society relationship. An important approach of health social work is putting policy into practice, and although existing social determinants of health are strongly related to governmental economic policies, whether social work can still maintain its professional value and practical approaches when the Chinese government is looking more towards social work to help it improve its policies (rather than challenge its authority and policy legitimacy) remains to be seen [24]. During the epidemic and post-epidemic era, the medical and health system, the emergency management system, and even the entire Communist Party and the government showed the characteristics of a “strong state” [53]. With the support of powerful information technology, the administrative system’s control has become particularly comprehensive, sensitive, and penetrating. The deep embedding of social work in the existing governance system has become a seemingly natural choice for the profession to improve its professional status. The question is, if social work becomes the capillary of state power in the social body, has its legitimacy been fundamentally questioned?

## 6. Conclusions

In this article, we have tried to explain how health social work, through the lens of legitimacy, can enhance its professional status in the context of the pandemic and be accepted and recognized by the government, clients, and other professionals. Although this study was conducted in the context of the epidemic in China, it is also relevant to the development of the international profession of social work, where the issue of legitimacy as a profession is always inevitable. Specifically, three dimensions of legitimacy should be sought by health social work: legality, value, and justification, which means that in daily practice, health social workers must take into account the political and policy contexts in which they practice, the social and practical values of their profession regarding health equity and justice, and the substantial support provided by government departments, community members, or other professions.

Policies, laws, and regulations guarantee the professional status of social work in the formal system, and they should also be concretized into the practical space of social work. The social work profession itself should also be adept at seizing various fleeting windows of opportunity to translate macro policy into effective micro practice, using policy advocacy and policy practice to fully interact with policy subjects [54] and promote the clarity and normalization of policy support. Recent research points to a long period when social workers invested a lot of energy into advancing policies that they thought would help the communities they worked with [55], which opens up more space for social work practice to be more specific and legalistic. Gal and Weiss-Gal provide specific practical pathways: proxy, recruitment networks, social work academia, civil society, and “insiders” [56]. Social workers need to understand and know that policy practice can take place at multiple levels: community, city, provincial (state), national, and any level that is important. For example, in China, social workers often complain that they do not have the support of the local government when working in the community, as they can work with knowledgeable people within the local government through community mobilization or lobbying to promote policies that benefit public health as well as social work.

Although expertise is considered “routine” within social work, its unique value and knowledge often cannot be reflected or transferred to outsiders (e.g., other professionals, clients, the public) [57]. The standardization of professional service techniques and professional competence with effective discretion are important steps for social workers to demonstrate their professional value. Health social work should emphasize the use of evidence-based practice, as well as the construction of specific logical models. Evidence-based practice can help social workers to obtain evidence about interventions and open up avenues of communication with professions such as medicine and psychology, rather than using their unique terminology and knowledge [58]; and logical models can help social welfare participants reflect together on the various processes and aspects of their work [59]. Second, professionalization also needs to further emphasize the establishment of value leadership and critical thinking in professional education. The current social work education system lacks the cultivation of professional values and the sense of social responsibility, and the influence of neoliberalism and managerialism makes social work services subordinate to theory and efficiency rather than the social equity and justice they claim [60,61], which makes social work students unable to effectively connect their professional knowledge with social reality, and unable to recognize where their professional values lie.

In its professional practice, the social work profession needs to constantly search for its advantages; actively participate in the community with relative autonomy and equal collaboration; find the parts that complement the existing system and the blind spots of the existing system; and further play its role in social welfare, the protection of vulnerable groups, and the promotion of social justice to reach the further legitimization and enhancement of the status of the social work profession. Although social work in China was traditionally introduced by the state, in this case, social work was already in action and closely integrated with the community before the state “intervened”. Therefore, for countries and regions where social work is still in its early stages of development, it is of the utmost importance for the profession to be active and to gain the support of the widest possible community.

## 7. Limitations

This study included a certain number of interviewees, and their views and conclusions are representative and somewhat consistent with empirical intuitions in China. However, the use of targeted sampling and the limited number of cases to which the researcher had access during the epidemic limit the generalization of the findings beyond the study population and to other countries. The first limitation of the study was the sample, as all participants were interviewed voluntarily and may have failed to reflect the voices of others who declined to be interviewed. In addition, participants’ responses were potentially positively biased because of the possible negative consequences of their statements (e.g., dismissal from their current job organization, etc.). Finally, all interviews were conducted in Chinese. Although the researcher attempted to accurately translate their responses into English, some of the nuances of their experiences may have been overlooked.

Another limitation of the study and a potential problem is whether the increased legitimacy and importance of social work in public health will prove to be just a “flash in the pan” when crises such as epidemics are over, when epidemic prevention and control are normalized, and when people’s attention is shifted, and whether social work can still be considered a part of public health without a constant push from the external environment. The success cases described in this article came from the earliest outbreaks and severe provinces in China, where the government, as a bureaucracy, usually requires response time, requiring forces from the society and the community to organize for “self-help”, giving the profession time and space to function rather than the traditional government-led situation. As a result, the success of health social work may have been magnified. In other areas, this change may not be evident, or may not have even occurred. Because of the political environment in China, national policy implementation may take a longer time, and in the future, continued attention to the changing course of social work legitimacy in China will still be needed for a long time.

## Figures and Tables

**Table 1:** List of social workers, experts, and scholars who participated in the key informant interviews.

Interviewees	Code	Profiles
**Government Officials (*n* = 2)**	P1	Former department Director of the National Health Commission of the PRC
	P2	Former department Director of the Ministry of Civil Affairs of the PRC
**Academic Experts and Scholars (*n* = 3)**	A1	University Professor
	A2	University Associate Professor
	A3	University Associate Professor
**Heads of Social Work Organizations (*n* = 3)**	O1	Social work organization secretary-general
	O2	Social service centre Chairman
	O3	Social work organization secretary-general
**Experts from Social Work Associations (*n* = 1)**	I1	Social work association Commissioner
**Heads of Hospitals and Medical Social Work Departments (*n* = 6)**	HA1	Vice President of Hospital
	HB1	Director of Social Work Department
	HC1	Director of Social Work Department
	HD1	Director of Social Work Department
	HE1	Director of Outpatient Clinic
	HF1	Vice President of Hospital
**Frontline Social Workers (*n* = 6)**	HG1	Medical Social Worker
	HG2	Medical Social Worker
	HH1	Medical Social Worker
	HC2	Medical Social Worker
	CS1	Community Social Worker
	CS2	Community Social Worker

## Data Availability

The data are not publicly available due to institutional and privacy restrictions.
